# Machining of Fe-Based Amorphous Alloy Ribbons with Sub-50 Femtosecond Laser Pulses

**DOI:** 10.3390/mi17020214

**Published:** 2026-02-05

**Authors:** Tamas Somoskoi, Miklós Füle, Peter Gaal, Mate Karnok, Gergely Kovacs, Lazar Toth, Judit Budai, Veronika Hanyecz, Ibolya Zsoldos, Karoly Osvay

**Affiliations:** 1 Department of Optics and Quantum Electronics, University of Szeged, Dóm tér 9, H-6720 Szeged, Hungary; 2 ELI ALPS, The Extreme Light Infrastructure ERIC, Wolfgang Sandner u. 3, H-6728 Szeged, Hungary; mfule@titan.physx.u-szeged.hu (M.F.);; 3 Department of Experimental Physics, University of Szeged, H-6720 Szeged, Hungary; 4 National Laser-Initiated Transmutation Laboratory, University of Szeged, H-6720 Szeged, Hungary; 5 Department of Materials Science and Engineering, Széchenyi István University, H-9026 Győr, Hungary

**Keywords:** Fe-based amorphous alloys, laser ablation, laser cutting, femtosecond laser, heat affected zone

## Abstract

Fe-based metallic glasses are ideal candidates to be utilized in transformer cores owing to their outstanding soft magnetic properties. However, they are difficult to machine properly by conventional means due to their mechanical brittleness and poor thermal conductivity. Here, the cutting of Fe_91_–Si_4.5_–C_4.0_–Al_0.5_ amorphous alloy ribbons is reported with a sub-50 fs laser pulses. A systematic study is performed on local morphological and chemical composition changes to the machined edge in comparison to crystalline metals. It is shown that only the innermost 80 μm wide region of the cut edge shows any detectable modifications, which is much less than for continuous laser machining. Therefore, the proposed method is indeed a valuable approach to overcome the fine machining difficulties of metallic glasses.

## 1. Introduction

Amorphous alloys have a peculiar structure, with a short-range order on the nanometer scale and a long-range disorder. The lack of a crystalline structure makes them isotropic, as they do not have grain boundaries. These properties are similar to those of glasses; thus, they are often referred to as metallic glasses [1]. They possess a number of beneficial properties compared to crystalline materials due to the lack of isolation defects and grain boundaries [2]. These include high mechanical hardness (3–4 times harder and stronger than crystalline steel [3], high fracture strength, high elastic limit, corrosion resistance, and beneficial soft magnetic properties [1]). The latter is related to the fact that metals with disordered structures can respond more readily to changes in a magnetic field because of the lack of grain boundaries. Although most ferromagnetic materials have an irreversible non-linear response of magnetization to an imposed magnetic field [4], Fe-based amorphous alloys have negligible hysteresis at frequencies that are normally used in AC power equipment. This property is particularly valued as Fe-based amorphous core energy losses can be lower by 75% compared to crystalline iron [5,6]. Due to these properties, such materials have become the core interest of electric and automotive industries for their use in transformers and iron cores of electric motors, inductors, sensors, and magnetic shielding materials [2]. For such applications, the components must be cut evenly and have the smoothest edges possible. Uneven material boundaries are prone to generate eddy currents, which ultimately reduces the performance of transformers.

However, there are a couple of disadvantages to the glass-like structure. The large-scale atomic disorder facilitates electron scattering; thus, their resistivity is about 2–3 times higher than that of the corresponding crystalline variant. More importantly, amorphous materials are thermodynamically metastable. If the temperature or pressure exceeds a certain point, viscous flows may occur or the material can evolve into a crystalline form [1]. Crystallization by temperature rise is further enhanced by the very low thermal conductivity of Fe-based alloys resulting in the loss of many advantageous properties of the amorphous structure. Lastly, amorphous alloys are also known for their high brittleness, which poses another problem for precise mechanical machining, often leading to jagged edges, macroscopic cracks, tool wear, and high machining temperature, which, again, makes the material susceptible to crystallization [7].

An alternative to mechanical techniques is laser-assisted material machining. Lasers have found widespread applications in material processing, such as drilling, cutting, welding, cladding, heat treatment, etching, and scribing [8,9]. These are due to their many advantageous features over mechanical machining. They are able to cut complex shapes with minimal kerf. They are not affected by material hardness and the tool is not worn due to the non-contact nature of the process [10]. Reports on continuous wave CO_2_ laser cutting on Fe-based amorphous alloys show that a heat affected zone 150–300 μm wide emerges at the cut edge. This has a crystalline structure with various grain sizes depending on the distance from the edge and shows micro hardness properties different from those of the intact amorphous phase [11]. The cut edge was irregular, as the imprint of the uneven energy distribution of the beam profile. The machined area also shows signs of melt 200–300 μm from the edge, and the ribbon takes a wavy shape resulting from local mechanical stresses. These are further indicators of crystallization and hardening of the material [12]. Better results could be achieved using pulsed microsecond and picosecond pulses as shown by Quintana [13]. X-ray diffraction analysis proves that crystallization can be avoided in this case. However, some of the ejected molten material was resolidified around the crater, forming a rim. This effect was shown to be weaker in case of shorter pulses, suggesting that sub-ps pulse machining could yield more even edges.

Ultrashort laser pulses have the great advantage of lower ablation thresholds and well-defined and localized energy deposition, which effectively reduce the excessive heat load on the work piece [14,15,16]. This is because of the underlying driving mechanisms of evaporation, thermomechanical spallation, and/or phase explosion, depending on the experimental conditions [17,18,19,20,21]. As a result, the machined area is more even, nearly free of molten layers and burrs [22]. Thus, they are proved to be invaluable in many fields of material (micro)machining. Despite their beneficial properties and widespread application for crystalline materials, only a few reports deal with amorphous alloy cutting using femtosecond pulses. Good processing quality and ablation efficiency was already demonstrated in Zr-based targets [23,24]. It was also proven that such an operation does not induce material crystallization. This is important because the beneficial properties of the amorphous structure can only be preserved in this way.

Here, the results of femtosecond laser cutting of a particular Fe-based amorphous alloy, Fe_91_–Si_4.5_–C_4.0_–Al_0.5_ is presented. Mechanical and chemical modification of the material are explored at various laser pulse energies and pulse numbers with the use of scanning electron microscope and EDX machine. It is shown that there is an optimal parameter range for ultrashort-pulse machining, where the smoothest machined edges are achieved along with keeping chemical composition changes to the minimum.

## 2. Materials and Methods

For the experiments, we used the front end of the Terawatt Ti:Sa Laser, developed at the University of Szeged, Hungary. The system is based on chirped-pulse amplification scheme. Femtosecond pulses at 800 nm from a commercial Kerr-lens mode-locked Ti:Sa oscillator are temporally stretched to 40 ps by a four-pass single transmission grating arrangement. A 10 pass Ti:S amplifier pumped by a Nd:YLF laser enhances the pulse energy from a fraction of nJ to over 2 mJ. The amplifier is equipped with a pulse-picker and an acousto-optical dispersive programmable filter for precise dispersion control. The pulses are then temporally compressed by a bulk BK7 glass and a series of chirped mirrors, resulting in 100 Hz repetition rate laser pulses with a duration and energy of 28 fs and 1.5 mJ, respectively. The fluctuation of the pulse energy is less than 1% RMS, while the pointing stability is within 25% of the diffraction-limited spot size [25]. These are important factors for the repeatability of the ablated structures. The number of pulses were selected using a mechanical shutter. All measurements were carried out in ambient air, with only one exception: when the effect of ambient liquid on the machining quality was tested.

**Figure 1:**
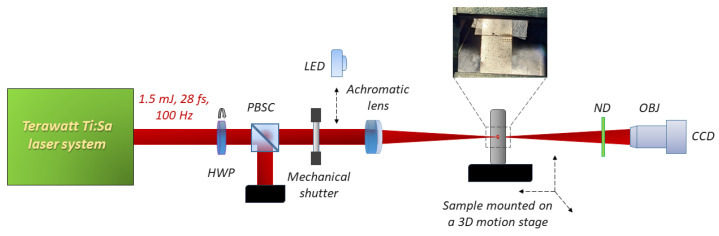
Schematic layout of the experimental setup. HWP—Half-Wave Plate; PBSC—Polarizing Beamsplitter Cube; ND—Neutral Density Filter; OBJ—Objective Lens.

The sample, an amorphous alloy ribbon (Fe_91_–Si_4.5_–C_4.0_–Al_0.5_) of 60 μm thickness, was vertically mounted on a 3D translator stage, as illustrated by Figure 1. The beam was focused on the target at normal incidence using an f = 25.4 mm biconvex achromatic lens. By passing through the lens, the pulses were stretched to 48 femtoseconds due to dispersion in the focusing elements. The precise determination of the focal spot was achieved through imaging with an objective lens, yielding 14.5 μm beam diameter (at 1/e^2^ peak intensity). The Gaussian peak fluence was calculated according to the ISO 21254-1:2025 standard [26] that is two times the pulse energy, divided by the area corresponding to 1/e^2^ beam diameter. Under this experimental conditions, the maximum fluence and intensity on the target was around 500 J/cm^2^ and 10^16^ W/cm^2^, respectively. Pulse energy adjustment was accomplished by the combination of a rotatable half-wave plate and a polarizer. The precise positioning of the target in the focal plane of the achromat was assisted by an external imaging system consisting of a LED light source and a camera. They were also used as an in situ diagnostic tool.

Point-like machining was performed by so-called percussion drilling, when the same spot is irradiated by multiple pulses. Linear cuts were achieved by line scan [20] with the use of a motorized translator stage. It was synchronized to the optical shutter, so the shutter was closed until the stage accelerated to the pre-defined cutting velocity, ensuring that each surface element was irradiated by the same number of pulses. To make a circular cut, a rotation stage was used in a manner similar to that described above.

Initial tests showed that single pulse irradiation barely induces any modification to the target—as seen through a scanning electron microscope—even at an extremely high fluence of 450 J/cm^2^. So we performed a series of multiple-shot tests on each target. The resulting mechanical and chemical changes were analyzed under various irradiation conditions. The sample morphology was inspected a posteriori by bright field and scanning electron microscopy (Scios 2 by Thermo Fisher Scientific, Waltham, MA, USA) as well as profilometry (Alpha-Step D-600 stylus profiler by KLA, Milpitas, CA, USA) to reveal the depth dimension. Electron diffraction spectroscopy (Quantax EDS by Bruker Corporation, Billerica, MA, USA) was used to analyze changes in the local composition of the material.

## 3. Results

### 3.1. Percussion Drilling

**Figure 2:**
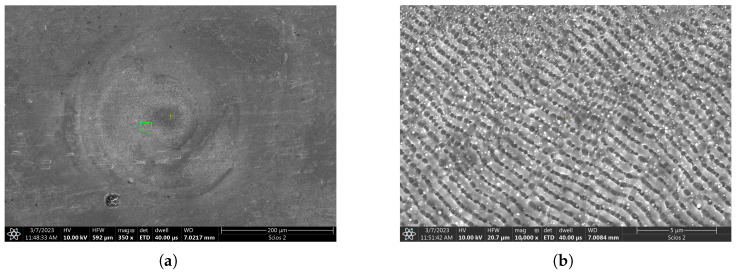
SEM image of a typical irradiation area exposed to 10 pulses at the 15 J/cm^2^ peak laser fluence. (**a**) Overview image showing the three broad regions with different morphologies. (**b**) Enlarged view of a rectangular area indicated on panel (**a**), showing characteristic LIPSS ripple features.

In this section, first some qualitative observations are made on the target in response to ultrashort pulse treatment before moving on to the full in-depth morphological analysis. Figure 1 shows a typical image of the target area that was irradiated by 10 pulses at the 15 J/cm^2^. Three broad regions can be identified around the interaction region based on SEM images as can be seen on Figure 2:

1. The central area has a smooth surface and roughly extends to about 25 μm. It corresponds in magnitude to the focal spot diameter (14.5 μm at 1/e^2^ peak intensity). This central area can only be identified at such relatively low fluences, since at large energies and/or higher number of pulses more severe morphological changes take place and this region becomes totally evaporated.
2. We can identify a rough, rim-shaped region around the central spot. The shape is less circular, and its diameter spans in the range of 180–250 μm. It has a distinctive feature of a highly modulated coarse surface. At higher magnifications, a periodic ripple structure can be uncovered. The ablation crater tends to partially overspread this region as well at high energies.
3. There is also a smooth circular outer region that surrounds the coarse area. This is more apparent at higher fluences, seen as a reflectivity change in the SEM image. It has no visible surface structure, only a characteristic bright outer contour line Later in the text, it will be referred to it as the heat affected zone (HAZ) and an explanation will be given on the origin of this name.

As mentioned above, a periodic ripple structure can be observed close to the center of the beam after irradiating with a few tens of pulses. Similar periodic features have been observed for numerous crystalline materials, metals, semiconductors, and dielectrics alike. This phenomenon occurs in the case of polarized irradiation, close to the ablation threshold and is referred to as Laser-Induced Periodic Surface Structures (LIPSS) [27,28]. The observed spatial periodicity varies around 850 nanometers in our case, which closely matches the laser wavelength. This subclass is termed low spatial frequency LIPSS (LSFL). According to the most widely accepted explanation, these can be interpreted as the result of an interference effect between the incident and the reflected waves, which induces periodic material removal [29,30]. In addition, hydrodynamic reorganization of a molten layer was also proposed as the driving mechanism [31].

**Figure 3:**
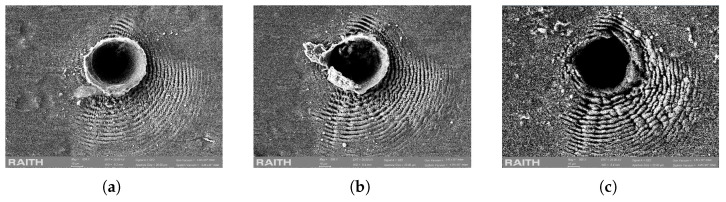
SEM images showing the evolution of the ablation crater at an increasing number of incident pulses at a 100 Hz repetition rate: (**a**) 60 pulses, (**b**) 125 pulses, (**c**) 500 pulses.

With an increasing number of pulses (50–100), a hole starts to be bored. Some of the ejected material re-deposits around the ablation crater and creates a loosely bound funnel structure. Subsequent pulses tend to disintegrate the crater rim, leaving only the circular opening behind on a flat surface and some of the remaining ripple structure. A similar effect was reported in the case of the crystalline steel sample [17]. Figure 3 shows the effect of 60, 125, and 500 pulses on the test site, respectively.

**Figure 4:**
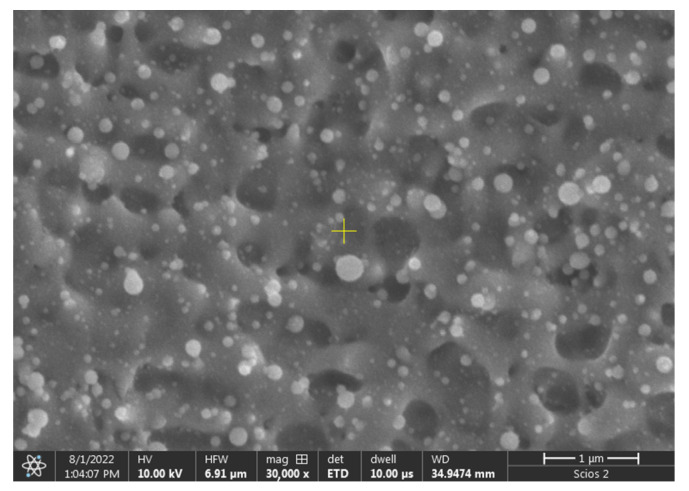
SEM image within the second region showing spherical-shaped nanostructures at high magnification.

In the vicinity of the ablated area sub-micron-sized spherical structures can be identified on Figure 4. These are probably resolidified ejected liquid droplets (also known as spherical nanoparticles). Similar nanostructures were observed for crystalline metal targets [17,18,32,33]. Their presence indicates that the ablation mechanism is at least partially thermally driven.

**Figure 5:**
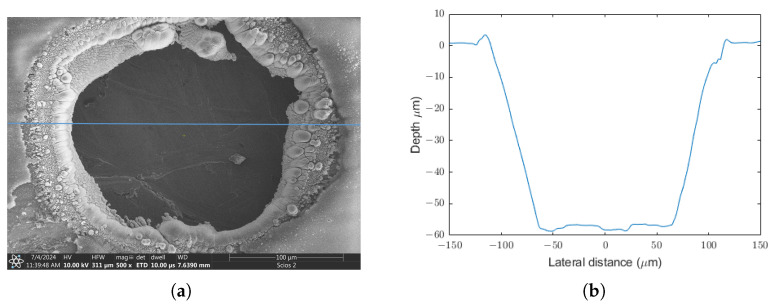
(**a**) SEM image of a through-hole ablation crater produced by 1000 pulses at 110 J/cm^2^ peak fluence. A horizontal line shows the scanning direction of the profilometer. (**b**) Corresponding depth profile of the above region. The bottom of crater is almost flat because the ribbon was perforated in this experiment. Note that the bottom of the crater looks uneven because it is the surface of a base plate supporting the sample.

**Figure 6:**
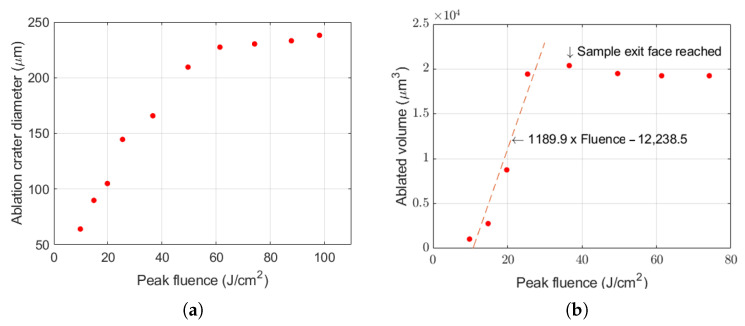
Evolution of the ablated area at 1000-on-1 testing regime. (**a**) Diameter of the crater at the entrance face as a function of peak fluence. (**b**) Ablated volume versus laser fluence. The curve saturates at a point where the sample is fully perforated. The linear segment is fitted by a dashed line.

To obtain a quantitative description of the ablation characteristics, a series of systematic measurements were performed over a wide fluence range in order to establish the onset of material removal and the optimal parameter range for processing. For this, the number of pulses that interact in the same spot was set to 1000 pulse. The laser peak fluence was varied between 10 and 110 J/cm^2^. A stylus profilometer was utilized to obtain information about the depth profiles of the ablation craters and the topography of the surrounding area. A line scan was performed along one axis of all irradiated areas. The scanning axis and the obtained cross section are illustrated in Figure 5:

The diameter of the ablation craters can be deduced from the profilometry line scan. Figure 6 shows a linear increase with laser fluence until the maximum crater size saturates at around 60 J/cm^2^. The surface of the sample outside the crater is flat and free from molten burrs and debris.

The volume of the ablated material can also be estimated by approximating the craters with cylindrical symmetry. A quasi-linear dependence of the ablated volume versus the laser fluence was observed in the low fluence range. This tendency holds until the laser cuts through the ribbon target, as can be seen on Figure 6. From that point on, the ablated volume cannot be increased further. This saturation behavior is shown on the graph above the 30 J/cm^2^ fluence regime. Extrapolating the linear line segment to zero implies that there would be no material removal below that certain fluence. The experimental results yield a value of 10.8 J/cm^2^. We consider this as an estimate for the ablation threshold in case of 1000 pulse irradiation. It is well known that the ablation threshold tends to decrease with pulse number due to various accumulation effects. There is an empirical rule that is commonly used in the literature to model this effect [34]:

(1) $$F_{th}\left(N\right)=F_{th}\left(1\right)N^{S-1}$$

In Equation (1) *F*_th_(*N*) represents the threshold fluence for *N*-pulse irradiation, while S is a constant, called the incubation parameter. The value of S ranges from 0.8 to 0.9, depending on the type of material [35]. To the best of our knowledge, this parameter has not yet been determined for Fe-based amorphous alloys. However, we can use the values of the upper and lower parameters (0.8 and 0.9) to set the limits for the ablation threshold in case of single-pulse irradiation. By substituting *F*_th_(1000) and S into Equation (1), one will obtain 20–40 J/cm^2^ for the single shot threshold, depending on the S parameter. Therefore, the single shot threshold would be 2–4 times higher, compared to the net effect of 1000 pulses.

**Figure 7:**
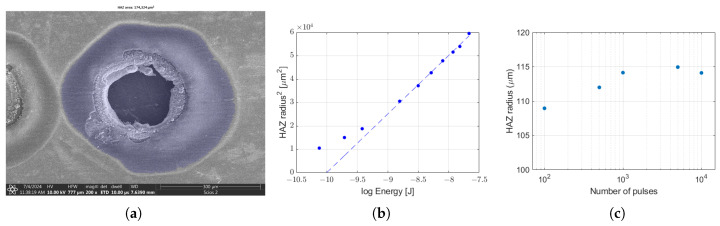
Spatial extent of the heat affected zone (HAZ) at various experimental parameters. (**a**) SEM image of the same crater depicted on Figure 5a at a larger field of view. The HAZ region—extracted by image evaluation—is highlighted in blue. (**b**) Square of the HAZ radius as a function of laser fluence. (**c**) Area of the heat affected zone at different number of incident pulses.

It was mentioned before that there is a darker area that surrounds the ablation crater, which is referred to as the heat affected zone (HAZ). The spatial extent of the HAZ was determined by image evaluation algorithms applied to SEM images These include bilateral filtering for edge-preserving noise removal, flood-fill segmentation, and morphological closing operations. The characteristic diameter of the HAZ region was obtained by approximating the area with a circle. This hypothetical circle was selected to have an area equivalent to the HAZ region. The selected region is shown in Figure 7 below.

The square of the heat affected zone radius shows a linear behavior as a function of the logarithm of pulse energy, as indicated by the middle panel of Figure 7. This can be interpreted on the basis of a simplified 3D thermal diffusion model when a point source acting on a semi-infinite solid [36]. Equation (2) describes the evolution of local temperature increase as a function of the radial coordinate (*R*) and time (*t*):

(2) $$\Delta T=\frac{2Q}{c\rho {\left(4\pi at\right)}^{\frac{3}{2}}}e^{-\frac{R^{2}}{4at}}$$

The pulse energy is denoted by *Q*, while *c*, *ρ* and *a* stand for the following material parameters: specific heat capacity, mass density and thermal diffusivity, respectively. By rearranging the equation, we arrive at the following formula (Equation (2)):

(3) $$R^{2}\left(Q,t\right)=4atlog\left(Q\right)+b\left(t\right)$$

Here, the second term, *b*, is a function of time and material parameters. Such dependence is visible on Figure 5b. This implies that the dark area of the SEM images—called ‘HAZ’ before—is indeed of thermal origin. The model neglects the spatial extent of the source as compared to the investigated area for ease of computation. It is also visible from the graph that the lowest three points do not follow well the linear tendency. This is because the point source assumption loses its validity as we approach smaller spatial regions. This happens when the size of the source (focal spot) becomes comparable to that of the heat affected zone. In addition, this simple model also assumes that the material parameters are constant and unaffected by the local change in temperature and pressure, which is not realistic. However, the area of the HAZ region was mostly unaffected by the number of incident pulses over two orders of magnitude at a 100 Hz repetition rate, as illustrated by Figure 7. This is in accordance with the thermal model and can be explained because the characteristic time of heat diffusion is much less than the pulse separation time (thermal diffusion for amorphous steel is <1 mm^2^/s).

### 3.2. Cutting

**Figure 8:**
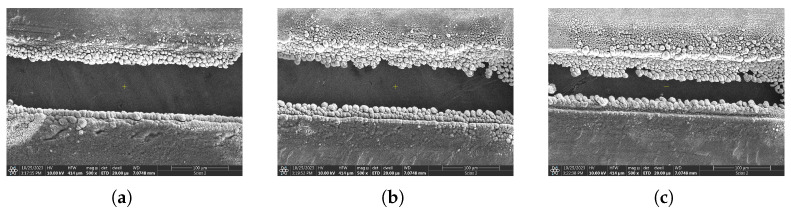
SEM images showing the effect on linear cutting at various laser fluences: (**a**) 78.0 J/cm^2^, (**b**) 62.4 J/cm^2^, (**c**) 42.2 J/cm^2^.

The sample was cut along a line by moving the target in the lateral direction at constant speed of 1 mm/s with respect to the optical axis. In the experiments, only single-pass cuttings were tested. Initial line scanning tests showed that better quality machined edges could be achieved at higher fluences. At lower energies the machined material boundary tends to be less even, as can be seen on scanning electron microscopic images in Figure 8 below. On the other hand the heat affected zone also increases with fluence following the same trend as in case of percussion drilling (see Figure 7).

**Figure 9:**
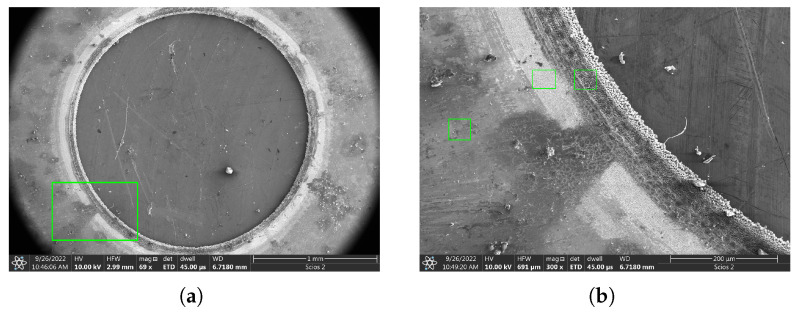
SEM image of the amorphous ribbon sample subject to circular cutting by continuous rotation of the target. (**a**) Overview image of the whole machined area. (**b**) Enlarged view of a rectangular area indicated on panel (**a**), showing various surface features along the rim. Three regions were highlighted, where the target was sampled by EDX microscopy. These are referred to as the ‘Intact region’, ‘HAZ region’, and ‘Edge region’.

For a continuous circular cut, the target was rotated by a motorized stage at a constant tangential velocity of 1 mm/s while it was irradiated at a pulse repetition rate of 100 Hz. The applied fluence was 110 J/cm^2^, and the workpiece was moved in a single revolution. Different rotation speeds were examined, but the above test condition showed an adequate result in terms of edge quality. A 100–150 μm broad modified area could be observed in the SEM images of Figure 9. Within this region, a roughly 80 μm-wide inner rim shows more severe morphological changes, with cone-like protruded micro-features. Schille [37] reported similar structures by crystalline steel and copper samples after treatment with 100–250 fs laser pulses.

**Figure 10:**
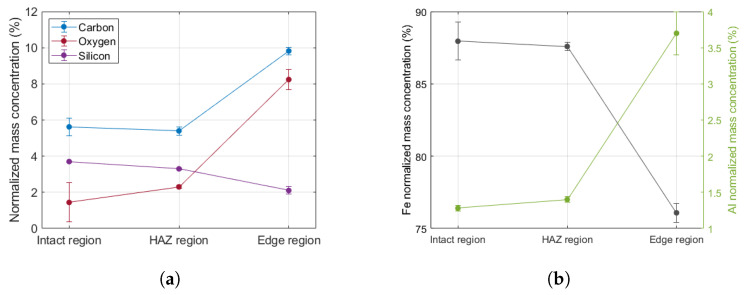
Relative material composition at different distances from the cut boundary: (**a**) Relative composition of carbon, oxygen, and silicon. (**b**) Relative iron and aluminum content.

Energy-dispersive X-ray spectroscopy (EDX) was also performed to investigate the alteration in material composition triggered by the interaction with ultra-short laser pulses. This analysis shows that only the *Edge region* has a significantly different material composition as depicted by Figure 10. An enrichment in aluminum, carbon, and oxygen can be observed moving closer to the cut boundary, while the iron content drops. Material composition at the HAZ region is indistinguishable from the intact sample surface. The consistency of results are proven by the low error bars.

### 3.3. Comparison with Crystalline Metals

**Figure 11:**
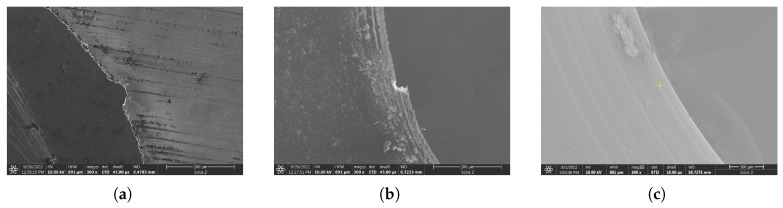
SEM image showing circular cut edges for different crystalline material targets: (**a**) 9 μm thick aluminum, (**b**) 20 μm thick aluminum, (**c**) 5 μm thick titanium.

Finally, a few comparative measurements were made by linear cutting of crystalline samples. Three types of foil targets were tested in total: 9 μm thick aluminum, 20 μm thick aluminum, and 5 μm thick titanium ribbons (please refer to Figure 11). The laser parameters and the experimental setup were kept the same that was used for the amorphous target to allow for an unbiased comparison. The most distinctive feature is the lack of spherical nano structures, which were present for the amorphous alloy. A HAZ was visible in all cases though, and is shown as dark rings at the boundaries through brightfield microscopy. However, it is more pronounced in the case of aluminum foils, at approximately 200 μm in width from the machined edge. The titanium target also shows signs of thermal modification, although in lesser extent of only 100 μm. There are also significant differences in the evenness of the machined rim of the two materials. The aluminum targets bear definite kerf along the edge and the shape is ruptured. Gaps of several tens of micrometers in size can be found. The flaws seen on the titanium target are much less; imperfections on the order of 1 μm can be seen in the higher magnification SEM images.

## 4. Discussion

In this paper, it was demonstrated that ultra-short laser-driven machining of Fe-based amorphous metallic alloys is a possible and promising approach. In this way, a quality similar to that for the traditional crystalline metals can be achieved in cut-edge evenness. This is a remarkable feature, since metallic glasses are known for their low thermal conductivity and mechanical brittleness. A thin boundary region of less than 100 μm was observed, which shows altered morphology and material composition. The latter can be attributed to oxidation as a result of the broken chemical bonds that occur as a result of ionization of the material. This is a significant improvement compared to CW laser machining, which results in a several hundred micrometer wide modified boundary region, where crystallization was also observed.

Numerical simulations on molecular dynamics have shown that there are multiple ablation mechanisms in the ultrashort laser interaction [19,20,38]. The presence of sub-micrometer spherical structures near the edge can be explained as resolidified liquid droplets. This indicates that the dominant ablation mechanism is phase explosion. This is the characteristic mechanism at high fluences, several times exceeding the ablation threshold. In contrast, thermomechanical spallation occurs in the low-energy regime. The main limitation of the mechanical machining of such ribbons is the uneven boundary of the machined part. This gives rise to eddy currents, which ultimately lower the efficiency of soft magnetic properties such as transformer cores. Such behavior is highly unlikely for ultrashort laser processing beyond the above-mentioned modified edge region. Outside this region, a darker HAZ is formed. The size of this region is unaffected by pulse accumulation at 100 Hz but shows exponential growth with pulse energy. This behavior is characteristic of temperature fields induced by point sources on a semi-infinite material. The flatness and material composition of the HAZ area is indistinguishable from the intact area of the ribbon. However, the zone of thermal impact is of great importance because metallic glasses are metastable and tend to revert to crystalline structures if exposed to high temperatures and pressures. Absolute verification can be performed by X-ray diffraction techniques.

An estimation was given of the ablation threshold, that is, the lowest fluence at which material removal occurs. The method was based on the combination of scanning electron microscopy and profilometric measurements. The dependence of crater volume on laser fluence was extrapolated to zero. Since our measurements were performed in a multishot testing regime (1000-on-1), accumulation effects should also be considered. Here, 10.8 J/cm^2^ was found for the multiple-shot ablation threshold, and the estimated single-shot threshold is within the limits of 20–40 J/cm^2^. Note that previously reported ablation thresholds for crystalline metals are much lower than this value. Values of 0.5–1.7 J/cm^2^ for a single shot and 0.04–0.33 J/cm^2^ for 1000-on-1 tests were observed for steel, aluminum, and copper [34,37,39]. This difference can be attributed to the distinct structure and material properties of metallic glasses.

Nevertheless, the evenness of the machined boundary region is similar to that of crystalline aluminum. Chemical compositional changes are limited to the very edge of the sample (80 μm width), so the beneficial soft magnetic properties are preserved for the rest of the workpiece, reaching the ultimate goal of our study.

## 5. Conclusions

This paper systematically investigated how the properties of a special metallic alloy film with an amorphous structure at room temperature change under laser machining. The sample was exposed to 48 fs laser pulses to study three machining geometries experimentally: hole drilling, straight linear cutting, and circular cutting. EDX microscopic and morphological investigations revealed an 80 μm-wide edge along the irradiated area, characterised by uneven surface microstructures and chemical changes due to oxidation. However, the advantage of ultrashort pulses is apparent against the 150–300 μm wide molten region reported for continuous wave lasers. A heat-affected zone was also observed surrounding the edge region, but the material composition and surface flatness were indistinguishable from those of the native material. This strongly implies that laser treatment does not affect the soft magnetic properties. We believe that our results contribute to the use of this material, which has very small hysteresis due to it being a transformer core material. Further research will focus on improving the machining quality by using more advanced beam delivery techniques, such as galvanometer scanning.

## Data Availability

The datasets used and analyzed during the current study are available from the corresponding author on reasonable request. Correspondence and requests for materials should be addressed to Tamás Somoskői (tamas.somoskoi@eli-alps.hu).

## Abbreviations

The following abbreviations are used in this manuscript:

LIPSS	Laser-induced periodic surface structures
EDX	Energy-dispersive X-ray spectroscopy
HAZ	Heat affected zone
SEM	Scanning electron microscope
